# Treatment outcomes of advanced hepatocellular carcinoma in real‐life practice: Chemotherapy versus multikinase inhibitors

**DOI:** 10.1002/cam4.5224

**Published:** 2022-09-09

**Authors:** Songporn Oranratnachai, Sasivimol Rattanasiri, Ekaphop Sirachainan, Amarit Tansawet, Nilubol Raunroadroong, Gareth J. McKay, John Attia, Ammarin Thakkinstian

**Affiliations:** ^1^ Department of Clinical Epidemiology and Biostatistics, Faculty of Medicine Ramathibodi Hospital Mahidol University Bangkok Thailand; ^2^ Oncology Clinic, Sriphat Medical Center, Faculty of Medicine Chiang Mai University Chiang Mai Thailand; ^3^ Division of Medical Oncology, Department of Medicine, Faculty of Medicine Ramathibodi Hospital Mahidol University Bangkok Thailand; ^4^ Department of Surgery, Faculty of Medicine Vajira Hospital Navamindradhiraj University Bangkok Thailand; ^5^ Chemotherapy Division Lampang Cancer Hospital Lampang Thailand; ^6^ Centre for Public Health, School of Medicine, Dentistry and Biomedical Sciences Queen's University Belfast Belfast UK; ^7^ Centre for Clinical Epidemiology and Biostatistics, School of Medicine and Public Health, Hunter Medical Research Institute University of Newcastle New Lambton New South Wales Australia

**Keywords:** FOLFOX, hepatocellular carcinoma, multikinase inhibitors, real‐world data, Sorafenib

## Abstract

**Background:**

Multikinase inhibitors (MKIs) represent the main treatment options for advanced hepatocellular carcinoma (aHCC). However, accessibility in developing countries is limited. A chemotherapy, Fluorouracil and Oxaliplatin (FOLFOX), offers a less expensive treatment. Therefore, this study sought to compare the clinical effectiveness of FOLFOX with Sorafenib as a first‐line treatment for aHCC in real‐life practice.

**Methods:**

A retrospective aHCC cohort from four Thai hospitals was investigated for patients who received FOLFOX or Sorafenib between 2013–2019. Multiple imputation by chained equations addressed missing covariate data in a treatment effect model using Weight‐adjusted‐censoring inverse‐probability‐weighted regression adjustment; overall survival (OS) and progression‐free survival (PFS) were estimated.

**Results:**

A total of 504 patients were included, (Sorafenib [*n* = 382] and FOLFOX [*n* = 122]). The treatment effect model estimated a median OS for Sorafenib and FOLFOX of 11.38 and 8.22 months, representing a significantly shorter OS (95% confidence interval) of −3.16 (−6.21, −0.11) months for FOLFOX, *p* = 0.042. A significant shorter median PFS of FOLFOX to Sorafenib of −2.13 (−3.03, −1.24) months, *p* < 0.001, was reported.

**Conclusion:**

Despite significantly shorter median OS and PFS than Sorafenib, FOLFOX still extended OS by 8.22 months. This evidence may offer clinical utility to physicians considering treatment options for aHCC in low resource settings.

## Graphical abstract

Hepatocellular carcinoma treatment in limited resource countries was limit due to cost. Real‐world evidence reflects clinical effectiveness in general practice. Even though FOLFOX had significant shorter survival than Sorafenib, but FOLFOX still extended median overall survival by 8.2 months.

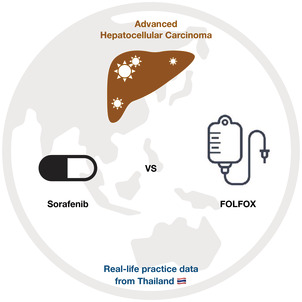

## 1 INTRODUCTION

Liver cancer is the third most common cause of cancer related death and ranks sixth in incident cases worldwide.^1^ With a 5‐year survival rate of 18%, liver cancer is the second most lethal malignancy, after pancreatic cancer.^2^ The common risk factors for hepatocellular carcinoma (HCC) are chronic hepatitis B virus (HBV) or hepatitis C virus (HCV) infections and liver cirrhosis from any cause. HCC treatment is dependent on the Barcelona Clinic Liver Cancer (BCLC) stage^3^ which accounts for liver function, patient's performance status incorporating tumor‐related symptoms, and tumor burden. Early stage patients commonly require localized treatments, compared to those with more advanced disease with preserved liver function, that necessitate multikinase inhibitors (MKI); supportive care is provided to patients with additional liver dysfunction.

Sorafenib was the first MKI approved by the Food and Drug Administration (FDA) as a treatment option for advanced stage HCC and has become the standard of care for frontline therapy. Overall survival (OS) is a universally accepted direct measure of clinical benefit that represents the duration of patient survival from the time of treatment initiation. Progression‐free survival (PFS) is a direct or surrogate measure of clinical benefit representing the time from treatment initiation until disease progression or worsening. Findings from a pivotal HCC trial^4^ showed that treatment with Sorafenib increased median OS from 7.9 to 10.7 months. Other MKIs, including Brivanib,^5^ Sunitinib,^6^ and Linifanib^7^ subsequently failed to significantly improve OS in comparison, until Lenvatinib was approved by the FDA in 2018 following a non‐inferiority trial^8^ demonstrating anti‐tumor activity with a median OS of 13.6 months.

Although Sorafenib and Lenvatinib are considered standard treatments for intermediate and advanced HCC in western countries,^4^, ^9^, ^10^ accessibility to these drugs is still limited in developing countries including Thailand, due to their cost. As such, chemotherapy is an alternative systemic treatment for HCC in developing countries. Evidence from a phase 3 study of a combination of Fluorouracil, Leucovorin, and Oxaliplatin (known as FOLFOX regimen) and Doxorubicin showed benefits in PFS, although not OS.^11^ To date, no study has directly compared the efficacy of MKIs and chemotherapy; this would provide evidence of clinical effectiveness in resource limited settings.

As Sorafenib is the recommended treatment option for advanced HCC according to most international treatment guidelines,^10^, ^12^, ^13^ many patients who meet the necessary recommended criteria are financially reimbursed, and can access Sorafenib as a first‐line systemic treatment. Conversely, patients who are unable to access Sorafenib due to financial or economic constraints, or with borderline abnormal liver function, would instead receive FOLFOX as a first‐line treatment. Although previous network meta‐analysis of randomized controlled trials (RCT)^14^ showed that Lenvatinib followed by Sorafenib could best prolong OS, FOLFOX may represent a viable alternative, especially where access is constrained. As such, this study was undertaken to compare the clinical effectiveness of FOLFOX relative to Sorafenib in advanced HCC patients using real world data.

## 2 MATERIALS AND METHODS

A multicenter retrospective cohort of advanced HCC patients were recruited from four study hospitals, i.e., Ramathibodi Hospital, Maharaj Nakorn Chiang Mai Hospital, Lampang Cancer Hospital, and Vajira Hospital. Medical records between January 2013 to December 2019 were screened and patients were included if the following inclusion criteria were met: aged 18 years or older, pathologically or clinically confirmed HCC diagnosis (BCLC stage A/B after failure of local treatment or BCLC stage C), and received any first‐line systemic therapy including Sorafenib or Oxaliplatin‐based chemotherapy (FOLFOX). Patients whose medical records were not available for review were excluded.

Demographic, clinical, radiological, and laboratory data were retrospectively collected and reviewed. HCC diagnosis and treatment decisions were made by physicians at each study hospital in light of health insurance coverage or patient willingness to pay. Treatment details and response to treatment, defined by radiography, were collected and categorized according to Response Evaluation *Criteria* in Solid Tumors version 1.1.^15^

Treatments of interest included Sorafenib and FOLFOX. Sorafenib was administered by daily dose until disease progression while FOLFOX was intravenously infused fortnightly until either disease progression or unaccepted toxicity was reached. Dosage for both drugs was adjusted by primary physicians in each hospital according to patients' performance status and comorbidities.

OS was the primary outcome of interest defined as time from treatment initiation until death from any cause. A secondary outcome was PFS, defined as the time from treatment initiation to progression of disease or death. Patients' status (i.e. alive or dead) was verified by death certificate from the Ministry of Interior up to 31st December 2020. Disease control rates (DCR) were defined as a complete response, partial response, or stable disease as their best response. Adverse events (AEs) of interest were classified according to Common Terminology Criteria for Adverse Events version 5.0.^16^

The study protocol was approved from Ethics Committee of all study centers before starting data collection and management. (MURA2020/1317 for Ramathibodi Hospital, No.420/2020 for Maharaj Nakorn Chiang Mai Hospital, No.30/2564 for Lampang Cancer Hospital, and COA 198/2563 for Vajira Hospital).

### 2.1 Statistical analysis

Baseline characteristics were described by frequency and percentage for categorical variables and mean and standard deviation or median and range for continuous variables as appropriate. These characteristics were compared between FOLFOX and Sorafenib groups using Chi‐square or Fisher's exact test for categorical variables and student *t*‐test or Kruskal‐Wallis test for continuous variables, as appropriate.

Missing data was assumed as missing at random (MAR) and imputation using the Multiple Imputation Chained Equation (MICE) was performed. Logit, multi‐logit, and interval‐regression equations were used to impute binary, categorical, and continuous variables, respectively (see Table S1). The number of imputations (*n* = 50) was set to cover the highest fraction of missing information.

Non‐parametric Kaplan–Meier survival probabilities were estimated for OS and PFS by treatment groups. The treatment effect model by weight‐adjusted‐censoring inverse‐probability‐weighted regression adjustment (WAC‐IPWRA) was applied to estimate median OS and PFS for each treatment. Three models were constructed, i.e., treatment assignment, outcome, and censored models. For a treatment model, logistic regression was used to identify predictive factors associated with treatment assignment. For censored and outcome models, a parametric survival analysis with appropriate survival distribution according to the lowest Akaike information criterion was used to identify predictive factors. For each model, an initial univariate regression analysis was performed for each demographic, clinical, and baseline laboratory variable. Subsequently, a multivariate model with backward elimination was used to select significant co‐variables in each model. The conditional independence assumption, overlap assumption, and correct adjustment for censoring assumption were evaluated. DCR and AEs (any grade and grade ≥ 3) were described as the number of patients affected and percentage by treatment groups. All statistical analysis was undertaken using Stata software version 16 (Stata Crop). A *p*‐value <0.05 was considered significant.

## 3 RESULTS

A total of 504 patients were enrolled, with 382 patients receiving Sorafenib and 122 receiving FOLFOX. Baseline characteristics differed significantly between both treatment groups, see Table 1. Patients from the FOLFOX group were generally sicker than those from the Sorafenib group: they were more likely to be treated in a Northern regional hospital, through a universal health coverage or Social Security Scheme, had a BCLB stage C at diagnosis, or more likely to have a Child‐Pugh B/C classification, present with major vascular involvement (MVI), received previous local treatment, have had abnormal liver function including alkaline phosphatase, aspartate aminotransferase (AST), and alpha‐fetoprotein (AFP) compared to those from the Sorafenib group; these patients were also more likely to be younger and in receipt of fewer systemic treatments than the Sorafenib group.

**TABLE 1:** Participant baseline characteristics

Characteristics	Sorafenib (*n* = 382)	FOLFOX (*n* = 122)	*p*‐value
Region			
North (CM + LPCH)	182 (47.6)	90 (73.8)	<0.001
Central (RAMA+VH)	200 (52.4)	32 (26.2)	
Age, years (mean ± SD)	62.2 ± 11.7	55.4 ± 9.7	<0.001
Gender			
Male	327 (85.6)	108 (88.5)	0.414
Female	55 (14.4)	14 (11.5)	
Health coverage scheme			
UC + SSS	11 (2.9)	91 (74.6)	<0.001
CSMBS+Self‐pay	371 (97.1)	31 (25.4)	
Underlying disease			
Hepatitis B infection			
No	108 (28.3)	32 (26.2)	<0.001
Yes	249 (65.2)	67 (54.9)	
Unknown	25 (6.5)	23 (18.9)	
Hepatitis C infection			
No	244 (63.9)	52 (42.6)	<0.001
Yes	76 (19.9)	32 (26.2)	
Unknown	62 (16.2)	38 (31.2)	
Alcoholic cirrhosis			
No	335 (87.7)	106 (86.9)	0.814
Yes	47 (12.3)	16 (13.1)	
Smoking status			
Never smoker	96 (25.1)	26 (21.3)	<0.001
Ever‐smoker	92 (24.1)	60 (49.2)	
Unknown	194 (50.8)	36 (29.5)	
Alcohol use			
Never drink	94 (24.6)	16 (13.1)	<0.001
Ever drink	132 (34.6)	82 (67.2)	
Unknown	156 (40.8)	24 (19.7)	
BCLC stage at diagnosis			
A	46 (12.5)	6 (4.9)	<0.001
B	160 (43.5)	33 (27.3)	
C	162 (44.0)	82 (67.8)	
ECOG‐PS			
0–1	230 (60.2)	98 (80.3)	<0.001
2–4	9 (2.4)	3 (2.5)	
Unknown	143 (37.4)	21 (17.2)	
Child‐Pugh classification			
A	328 (86.8)	64 (52.5)	<0.001
B or C	50 (13.2)	58 (47.5)	
MVI			
No	216 (56.7)	54 (44.3)	0.017
Yes	165 (43.3)	68 (55.7)	
EHS			
No	160 (42.0)	53 (43.4)	0.778
Yes	221 (58.0)	69 (56.6)	
Previous local treatment			
No	142 (37.3)	86 (70.5)	<0.001
Yes	239 (62.7)	36 (29.5)	
Total no. of treatment, Median (range)	1 (1–8)	1 (1–4)	0.001
Laboratory			
ALP			
<3× ULN	355 (93.9)	102 (83.6)	<0.001
≥3× ULN	23 (6.1)	20 (16.4)	
AST			
<3× ULN	244 (64.4)	51 (41.8)	<0.001
≥3× ULN	135 (35.6)	71 (58.2)	
ALT			
<3× ULN	356 (93.9)	112 (91.8)	0.410
≥3× ULN	23 (6.1)	10 (8.2)	
Creatinine, mg/dl			
<1.5	338 (95.8)	117 (98.3)	0.261
≥1.5	15 (4.2)	2 (1.7)	
Hemoglobin, g/dl			
<8.5	8 (2.1)	1 (0.8)	0.694
≥8.5	364 (97.9)	118 (99.2)	
White Blood Cell, /mm^3^			
<4000	34 (9.0)	7 (5.7)	0.251
≥4000	343 (91.0)	115 (94.3)	
Platelet, /mm^3^			
<75,000	23 (6.1)	2 (1.6)	0.056
≥75,000	354 (93.9)	120 (98.4)	
AFP, ng/ml			
<400	16 1 (42.2)	31 (25.4)	<0.001
≥400	140 (36.6)	72 (59.0)	
Unknown	81 (21.2)	19 (15.6)	

*Note*: Value are expressed as *n* (%) unless otherwise indicated. *p*‐value by Chi2 or fisher's exact test for categorical variables, student *t*‐test or Kruskal‐Wallis test for continuous variables.

Abbreviations: AFP, alpha‐fetoprotein; ALP, alkaline phosphatase; ALT, Alanine aminotransferase; AST, Aspartate aminotransferase; BCLC, Barcelona Clinic Liver Cancer; CM, Maharaj Nakorn Chiang Mai hospital; CSMBS, Civil Servant Medical Benefit Scheme; ECOG‐PS, Eastern Cooperative Oncology Group performance status; EHS, Extrahepatic spreading; FOLFOX, Fluorouracil, Leucovorin, and Oxaliplatin; LPCH, Lampang Cancer hospital; MVI, major vascular involvement; RAMA, Ramathibodi hospital; SD, standard deviation; SSS, Social Security Scheme; UC, universal health coverage scheme; ULN, upper limit of normal; VH, Vajira hospital.

Missing data was as high as 45.6% for smoking status, followed by alcohol use (35.7%), Eastern Cooperative Oncology Group performance status (32.5%), AFP (19.8%), HCV infection (19.8%), HBV infection (9.5%), and creatinine (5.8%), see Table 2. Other missing covariate data, i.e., BCLC stage at diagnosis, laboratory measures other than creatinine and AFP, Child‐Pugh classification, MVI, extrahepatic spreading, and previous local treatment were missing for <5% of study participants. Missing covariate data was imputed using 50 iterations using a MICE for inclusion in other analyses.

**TABLE 2:** Covariate standardization summary for overall survival

Covariates	Standardized differences^a^		Variance ratio^b^	
	Raw	Weighted	Raw	Weighted
FOLFOX versus Sorafenib				
Region				
Central (RAMA+VH)	−0.55376	0.001354	0.780071	1.000737
Health coverage scheme				
CSMBS+Self‐pay	−2.1664	0.11228	6.815216	0.871981
Child‐Pugh classification				
B or C	0.799485	−0.10586	2.176245	0.89561
Aspartate aminotransferase				
≥3× ULN	0.457137	0.062585	1.063223	1.022973

Abbreviations: CSMBS, Civil Servant Medical Benefit Scheme; FOLFOX, Fluorouracil, Leucovorin, and Oxaliplatin; RAMA, Ramathibodi hospital; ULN, upper limit of normal; VH, Vajira hospital.

^a^ The closer the weighted difference is to zero the better the standardization.

^b^ The closer the weighted ratio is to one the better the standardization.

A treatment assignment model was constructed with only four significantly associated covariates retained in the model, including region, health coverage scheme, Child‐Pugh classification, and AST, see Table S3. These co‐variables were well balanced after weighting, in which their standardized weight mean differences were close to zero, and variance ratios close to one in line with the conditional independence assumption, see Table 2. In addition, the densities of the probabilities for receiving each treatment were plotted for each co‐variable with overlapping assumptions that all patients had a positive probability for receiving each treatment (see Figure S1).

At the end of follow up (31st December 2020), 464 out of 504 patients had died with a median follow‐up time of 5.72 (range: 0.20 to 86.75) months. Kaplan–Meier survival curves were constructed (Figure S2) indicating unadjusted median OS (95% confidence interval [CI]) of 7.02 (5.93, 8.07) and 4.26 (3.51, 4.62) months for Sorafenib and FOLFOX, respectively. Applying WAC‐IPWRA adjusted co‐variables to treatment assignment and OS, increased the estimated median OS (95% CI) for Sorafenib and FOLFOX to 11.38 (9.70, 13.07) and 8.22 (5.66, 10.78) months, respectively. FOLFOX had significantly shorter OS than Sorafenib by −3.16 (−6.21, −0.11) months, *p* = 0.042, see Table 3.

**TABLE 3:** Potential outcome means estimates for overall survival and progression‐free survival by treatment group: Weight‐adjusted‐censoring inverse‐probability‐weighted regression adjustment with log‐normal survival distribution

Treatment	Median OS (95% CI, months)	Median PFS (95% CI, months)
Sorafenib	11.38 (9.70, 13.07)	5.46 (4.83, 6.09)
FOLFOX	8.22 (5.66, 10.78)	3.33 (2.72, 3.94)

Abbreviations: CI, confidence interval; FOLFOX, Fluorouracil, Leucovorin, and Oxaliplatin; OS, overall survival; PFS, progression‐free survival.

For PFS, 303 patients had disease progression while 182 patients died; 15 patients were still under follow‐up or continuing treatment at the close of the study period. Unadjusted PFS curve by Kaplan–Meier method was constructed by Sorafenib and FOLFOX groups (Figure S3) indicating longer PFS in Sorafinib than FOLFOX. The WAC‐IPWRA models provided adjusted median PFS values of 5.46 (4.83, 6.09) and 3.33 (2.72, 3.94) months for Sorafenib and FOLFOX, respectively. Again, FOLFOX had significant shorter median PFS by −2.13 (−3.03, −1.24) months compared to Sorafenib, *p* < 0.001, see Table 3.

Of the 504 patients, 355 patients were evaluated for response to treatment, with 149 patients not evaluated representing 25% and 42% of the Sorafenib and FOLFOX groups respectively (Table S4). DCR occurred in 31.1% of patients for Sorafenib and 21.3% for FOLFOX. AEs were considered by treatment group and overall any grade AEs were slightly higher for Sorafenib than FOLFOX (i.e., 55.2% vs. 47.5%; Table 4), although this was not significant (*p* = 0.14). Serious AEs, grade 3 or higher, was lower for Sorafenib than FOLFOX (10.5% vs. 15.6%) but again this was not significant (*p* = 0.13). More hematologic AEs were observed in the FOLFOX treatment group, along with nausea, vomiting and neuropathy, while patients in the Sorafenib group were more likely to suffer diarrhea and hand/foot skin reactions.

**TABLE 4:** Adverse event summary by treatment group

Adverse event	Sorafenib (*n* = 382)		FOLFOX (*n* = 122)	
	Any grade	Grade ≥ 3	Any grade	Grade ≥ 3
Overall adverse events	211 (55.2)	40 (10.5)	58 (47.5)	19 (15.6)
Hematologic adverse event				
Anemia	1 (0.3)	1 (0.3)	1 (0.8)	1 (0.8)
Neutropenia	7 (1.8)	1 (0.3)	27 (22.1)	12 (9.8)
Thrombocytopenia	5 (1.3)	1 (0.3)	8 (6.6)	1 (0.8)
Febrile neutropenia	0	0	3 (2.5)	3 (2.5)
Non‐ hematologic adverse event				
Diarrhea	74 (19.4)	10 (2.6)	13 (10.7)	2 (1.6)
Hand‐foot skin reaction	147 (38.5)	17 (4.4)	1 (0.8)	0
Hepatic failure	26 (6.8)	10 (2.6)	3 (2.5)	3 (2.5)
Nausea and vomiting	5 (1.3)	0	10 (8.2)	0
Neuropathy	0	0	6 (4.9)	0

*Note*: Value are expressed as *n* (%), Chi square test *p*‐value for difference between Sorafenib and FOLFOX was 0.138 and 0.127 for any grade adverse events and grade ≥ 3 adverse events, respectively.

Abbreviation: FOLFOX, Fluorouracil, Leucovorin, and Oxaliplatin.

## 4 DISCUSSION

This retrospective cohort of advanced HCC patients included real world data from 504 patients and compared relative treatment effects between Sorafenib and FOLFOX. The treatment effect comparisons using WAC‐IPWRA estimators identified significantly shorter OS and PFS of three and 2 months respectively for FOLFOX compared to Sorafenib, which had median OS and PFS values of 11.4 and 5.5 months, respectively. Furthermore, the FOLFOX DCR was also approximately 10% lower compared to that of Sorafenib. While overall AEs were almost 8% lower in the FOLFOX treatment group compared to Sorafenib, serious AEs were slightly higher in FOLFOX, although neither was significant.

Baseline demographic characteristics were comparable to previous studies, i.e. predominantly male (65%–90%) and middle‐aged (50–68 years).^4^, ^8^, ^9^, ^11^, ^17^, ^18^, ^19^ HBV infection is more common in studies from Asia‐Pacific countries^9^, ^11^, ^17^, ^19^, ^20^, ^21^ compared to those from western countries.^4^, ^18^, ^22^, ^23^ To the best of our knowledge, no direct comparisons between Sorafenib and FOLFOX have been published previously. A pivotal multi‐center trial only compared Sorafenib to placebo in western (SHARP study^4^) and Asia‐Pacific countries.^9^ Several additional large,^18^ small,^17^, ^19^, ^20^, ^24^ single‐arm cohorts reported Sorafenib treatment outcomes in advanced/unresectable HCC patients.^17^, ^19^, ^20^, ^24^ These studies reported median OS and PFS for Sorafenib treatment ranging from 5 to 13.6 and 2.8 to 5.5 months, respectively. Our findings support those from previous clinical trials for OS (i.e., 11.4 vs. 5–13.6 months) and PFS (i.e., 5.5 vs. 2.8–5.5 months), although grade ≥3 AEs from our study were much lower (10.5%) than previous findings^9^, ^17^, ^18^, ^19^, ^24^ (22%–47%). Sorafenib dosing regimens may differ significantly between real‐life practice and clinical trials. Dose escalation strategies (i.e., initiated with low dose, then increased if tolerable) are common, so some patients may not receive the full recommended dosage in real‐life practice. As a result, severe AEs may be less likely in contrast to clinical trials restricted to full dosing regimens that may lead to a higher number of severe AEs.

The median OS and PFS of FOLFOX from a phase 3 RCT^11^ were 6.4 and 2.9 months, respectively. Other evidence from a French advanced HCC cohort^23^ reported median OS of 15.7 and 5.4 months for Child‐Pugh class A and B respectively with corresponding PFS values of 6.7 and 2.9 months. Two prospective single‐arm cohorts that investigated Capecitabine and Oxaliplatin (XELOX) combination therapies in unresectable HCC from France^22^ and extrahepatic metastatic HCC following local treatment in China,^21^ showed median OS and PFS values of 9 and 4 months respectively. As expected, XELOX studies reported significantly better OS rates for Child‐Pugh class A than the more severe Child‐Pugh class B phenotype. Approximately half of the FOLFOX participants were a more severe phenotype (not Child‐Pugh class A), and the unadjusted median OS Kaplan–Meier estimate was only 4.3 months, a value similar to the 5.4 months previously reported by Coriat and colleagues for Child‐Pugh class B patients.^23^ However, following adjustment of the treatment effect model for significant covariates, including the Child‐Pugh classification, the median OS value for FOLFOX increased to 8.2 months, which was greater than previous RCT estimates.^11^

Our study had several strengths. Previous RCTs have compared MKIs to other MKIs or placebo, but there have been no direct comparisons with chemotherapy; this may be due to Sorafenib has been approved by the FDA as the standard first‐line therapy since 2007, which was before the EACH trial^11^ was conducted and published in 2013. As such, a direct head‐to‐head RCT treatment comparison of FOLFOX and Sorafenib efficacy is unlikely to happen and an observational design may be the only way to answer this question. Nevertheless, real world data is more prone to selection and confounding bias that are compounded by treatment assignment, necessitating appropriate adjustment to adequately assess clinical outcomes. Counterfactual approaches^25^, ^26^ have been used to emulate the data as if generated by RCT to provide improved causal treatment effect estimates. As such, we used WAC‐IPWRA models to address the confounding effects represented in participant baseline characteristics that are apparent following treatment assignment, and which influence the OS and PFS estimates. Our findings may provide useful clinical evidence to guide physicians prescribing treatments like FOLFOX to advanced HCC patients, when MKI treatments are either unaffordable or unavailable.

Given the nature of our retrospective cohort study design, we were unable to avoid the issue of missing data. Despite addressing this concern through MICE approaches which assumes missingness is completely at random, MAR, or not at random; each of which could not be determined by statistical means. However, we assumed that the missingness was more likely to be related to the available data observed, i.e., MAR, and so MICE imputation models were constructed based on clinical outcomes (i.e., OS and PFS) plus additional available covariate data making the MAR assumption more probable.^22^ Furthermore, we purposely did not include supportive treatment comparisons within this analysis, and therefore the potential benefits of FOLFOX over best supportive care cannot be considered further. However, adjusted median values for OS and PFS from treatment effect models have previously suggested comparable to better, OS and PFS outcomes, than supportive care or placebo in RCTs.^4^, ^9^, ^27^

In summary, this study used real world practice data with counterfactual analysis methods to simulate an RCT of clinical effectiveness between FOLFOX and Sorafenib in advanced HCC patients. Despite a significant 3 and 2 month reduction in median OS and PFS values for FOLFOX compared to Sorafenib, FOLFOX led to reasonable OS of 8.2 months, with no significant difference in the rate of AEs. This evidence may be especially useful for physicians considering potential treatment options for advanced HCC patients in resource limited settings.

## AUTHOR CONTRIBUTIONS

Songporn Oranratnachai: Conceptualization, study design, project management, data collection and management, data analysis, interpretation, Writing – initial draft, and contributed to every draft thereafter. Sasivimol Rattanasiri: Supervision, Writing – review & editing. Amarit Tansawet: data collection. Nilubol Raunroadroong: data collection. Ekaphop Sirachainan: Supervision, study design, data collection, interpretation, writing – critically review & editing. John Attia: Supervision, Writing – critically review & editing. Gareth J. McKay: Supervision, Writing – critically review & editing. Ammarin Thakkinstian: Supervision, study design, data analysis, Writing – critically review & editing.

## FUNDING INFORMATION

This research did not receive any specific grant from funding agencies in the public, commercial, or not‐for‐profit sectors.

## CONFLICT OF INTEREST

All authors declared no known conflict of interests or personal relationships that could have appeared to influence the work reported in this paper; except AT received grant from the National Research Council of Thailand (NRCT: N42A640323). The grant sponsor had no role in the design or conduct this study.

## ETHICAL APPROVAL STATEMENT

The study protocol was approved from Ethics Committee of all study centers. (MURA2020/1317 for Ramathibodi Hospital, No.420/2020 for Maharaj Nakorn Chiang Mai Hospital, No.30/2564 for Lampang Cancer Hospital, and COA 198/2563 for Vajira Hospital).

## Supporting information


Table S1
Table S2
Table S3
Table S4
Figure S1
Figure S2
Figure S3

## Data Availability

The data that support the findings of this study are available from the corresponding author upon reasonable request.
