# Supplementary material for: Treatment outcomes of advanced hepatocellular carcinoma in real‐life practice: Chemotherapy versus multikinase inhibitors
Source: Cancer Med. 2022 Sep 9;12(3):3046–53. doi: 10.1002/cam4.5224 (PMC9939209; doi:10.1002/cam4.5224)
Supplement: Supplementary file 1 — Table S1 Table S2 Table S3 Table S4 Figure S1 Figure S2 Figure S3 [file CAM4-12-3046-s001.docx]

**Supplementary Materials**

**Supplementary Table 1** Imputation models

**Supplementary Table 2** Covariate missing data by treatment groups

**Supplementary Table 3** Factors associated with treatment assignment: Univariate and multivariate logistic regression

**Supplementary Table 4** Response to treatment

**Supplementary Figure 1** Overlapping plot

**Supplementary Figure 2** Kaplan-Meier survival curve for overall survival by treatment regimens

**Supplementary Figure 3** Kaplan-Meier survival curve for progression-free survival by treatment regimens

**Supplementary Table 1** Imputation models

|  | **Predictors** | | | | | | | | | | | | | | | | | | | | | | | | **Model** |
| --- | --- | --- | --- | --- | --- | --- | --- | --- | --- | --- | --- | --- | --- | --- | --- | --- | --- | --- | --- | --- | --- | --- | --- | --- | --- |
|  | **Region** | **Age** | **Gender** | **HCS** | **HBV** | **HCV** | **Alc. cirrhosis** | **Smoking** | **Alcohol use** | **BCLC** | **ECOG** | **Child** | **MVI** | **EHS** | **Local treatment** | **ALP** | **AST** | **ALT** | **Creatinine** | **Hemoglobin** | **WBC** | **Platelet** | **AFP** | **Treatment** |  |
| **HBV** | √ | √ | √ | √ |  | √ | √ | √ | √ | √ | √ | √ | √ | √ | √ | √ | √ | √ | √ | √ | √ | √ | √ | √ | logit |
| **HCV** | √ | √ | √ | √ | √ |  | √ | √ | √ | √ | √ | √ | √ | √ | √ | √ | √ | √ | √ | √ | √ | √ | √ | √ | logit |
| **Smoking** | √ | √ | √ | √ | √ | √ | √ |  | √ | √ | √ | √ | √ | √ | √ | √ | √ | √ | √ | √ | √ | √ | √ | √ | logit |
| **Alcohol** | √ | √ | √ | √ | √ | √ | √ | √ |  | √ | √ | √ | √ | √ | √ | √ | √ | √ | √ | √ | √ | √ | √ | √ | logit |
| **BCLC** | √ | √ | √ | √ | √ | √ | √ | √ | √ |  | √ | √ | √ | √ | √ | √ | √ | √ | √ | √ | √ | √ | √ | √ | mlogit |
| **ECOG** | √ | √ | √ | √ | √ | √ | √ | √ | √ | √ |  | √ | √ | √ | √ | √ | √ | √ | √ | √ | √ | √ | √ | √ | logit |
| **Child** | √ | √ | √ | √ | √ | √ | √ | √ | √ | √ | √ |  | √ | √ | √ | √ | √ | √ | √ | √ | √ | √ | √ | √ | logit |
| **MVI** | √ | √ | √ | √ | √ | √ | √ | √ | √ | √ | √ | √ |  | √ | √ | √ | √ | √ | √ | √ | √ | √ | √ | √ | logit |
| **EHS** | √ | √ | √ | √ | √ | √ | √ | √ | √ | √ | √ | √ | √ |  | √ | √ | √ | √ | √ | √ | √ | √ | √ | √ | logit |
| **Local treatment** | √ | √ | √ | √ | √ | √ | √ | √ | √ | √ | √ | √ | √ | √ |  | √ | √ | √ | √ | √ | √ | √ | √ | √ | logit |
| **ALP** | √ | √ | √ | √ | √ | √ | √ | √ | √ | √ | √ | √ | √ | √ | √ |  | √ | √ | √ | √ | √ | √ | √ | √ | intreg |
| **AST** | √ | √ | √ | √ | √ | √ | √ | √ | √ | √ | √ | √ | √ | √ | √ | √ |  | √ | √ | √ | √ | √ | √ | √ | intreg |
| **ALT** | √ | √ | √ | √ | √ | √ | √ | √ | √ | √ | √ | √ | √ | √ | √ | √ | √ |  | √ | √ | √ | √ | √ | √ | intreg |
| **Cr** | √ | √ | √ | √ | √ | √ | √ | √ | √ | √ | √ | √ | √ | √ | √ | √ | √ | √ |  | √ | √ | √ | √ | √ | intreg |
| **Hb** | √ | √ | √ | √ | √ | √ | √ | √ | √ | √ | √ | √ | √ | √ | √ | √ | √ | √ | √ |  | √ | √ | √ | √ | intreg |
| **WBC** | √ | √ | √ | √ | √ | √ | √ | √ | √ | √ | √ | √ | √ | √ | √ | √ | √ | √ | √ | √ |  | √ | √ | √ | intreg |
| **platelet** | √ | √ | √ | √ | √ | √ | √ | √ | √ | √ | √ | √ | √ | √ | √ | √ | √ | √ | √ | √ | √ |  | √ | √ | intreg |
| **AFP** | √ | √ | √ | √ | √ | √ | √ | √ | √ | √ | √ | √ | √ | √ | √ | √ | √ | √ | √ | √ | √ | √ |  | √ | intreg |

AFP, alpha-fetoprotein; Alc. Cirrhosis, alcoholic cirrhosis; ALP, alkaline phosphatase; ALT, alanine aminotransferase; AST, aspartate aminotransferase; BCLC, Barcelona Clinic Liver Cancer stage at diagnosis; Child, Child-Pugh classification; ECOG, Eastern Cooperative Oncology Group Performance Status; EHS, extrahepatic spread; HBV, hepatitis B virus; HCS, health coverage scheme; HCV, hepatitis C virus; intreg, interval regression; logit, logistic regression; mlogit, multinomial logistic regression; MVI, major vascular involvement; WBC, white blood cell

**Supplementary Table 2** Covariate missing data by treatment groups

| **Characteristics** | **Total**  (n=504) | **Sorafenib**  (n=382) | **FOLFOX**  (n=122) |
| --- | --- | --- | --- |
| **Underlying disease** |  |  |  |
| HBV infection | 48 (9.5) | 25 (6.5) | 23 (18.9) |
| HCV infection | 100 (19.8) | 62 (16.2) | 38 (31.2) |
| **Smoking status** | 230 (45.6) | 194 (50.8) | 36 (29.5) |
| **Alcohol use** | 180 (35.7) | 156 (40.8) | 24 (19.7) |
| **BCLC stage at diagnosis** | 15 (3.0) | 14 (3.7) | 1 (0.1) |
| **ECOG-PS** | 164 (32.5) | 143 (37.4) | 21 (17.2) |
| **Child-Pugh classification** | 4 (0.8) | 4 (1.0) | - |
| **MVI** | 1 (0.2) | 1 (0.3) | - |
| **EHS** | 1 (0.2) | 1 (0.3) | - |
| **Previous local treatment** | 1 (0.2) | 1 (0.3) | - |
| **Laboratory** |  |  |  |
| **ALP** | 4 (0.8) | 4 (1.0) | - |
| **AST** | 3 (0.6) | 3 (0.8) | - |
| **ALT** | 3 (0.6) | 3 (0.8) | - |
| **Creatinine** | 29 (5.8) | 27 (7.1) | 2 (1.6) |
| **Hemoglobin** | 11 (2.2) | 8 (2.1) | 3 (2.5) |
| **WBC** | 5 (1.0) | 5 (1.3) | - |
| **Platelet** | 5 (1.0) | 5 (1.3) | - |
| **AFP** | 100 | 81 (21.2) | 19 (15.6) |

Value are expressed as n (%)

AFP, Alpha-fetoprotein; ALP, Alkaline Phosphatase; ALT, Alanine aminotransferase; AST, Aspartate aminotransferase; BCLC, Barcelona Clinic Liver Cancer; ECOG-PS, Eastern Cooperative Oncology Group performance status; EHS, extrahepatic spreading; FOLFOX, Fluorouracil, Leucovorin, and Oxaliplatin; HBV, hepatitis B virus; HCV, hepatitis C virus; MVI, major vascular involvement; WBC, White Blood Cell

**Supplementary Table 3** Factors associated with treatment assignment: Univariate and multivariate logistic regression

| **Covariables** | **Univariate analysis** | | **Multivariate analysis** | |
| --- | --- | --- | --- | --- |
|  | **OR (95% CI)** | **P-value** | **OR (95% CI)** | **P-value** |
| **Region** |  |  |  |  |
| North (CM+LPCH) | 1 |  | 1 |  |
| Central (RAMA+VH) | 0.324 (0.206, 0.508) | < 0.001 | 0.212 (0.098, 0.457) | < 0.001 |
| **Age**, years | 0.948 (0.930, 0.967) | < 0.001 |  |  |
| **Gender** |  |  |  |  |
| Male | 1 |  |  |  |
| Female | 0.771 (0.412, 1.441) | 0.415 |  |  |
| **Health coverage scheme** |  |  |  |  |
| UC+SSS | 1 |  | 1 |  |
| CSMBS+Self-pay | 0.010 (0.005, 0.021) | < 0.001 | 0.006 (0.003, 0.015) | < 0.001 |
| **Underlying disease** |  |  |  |  |
| HBV infection |  |  |  |  |
| No | 1 |  |  |  |
| Yes | 0.802 (0.501, 1.285) | 0.359 |  |  |
| HCV infection |  |  |  |  |
| No | 1 |  |  |  |
| Yes | 2.148 (1.270, 3.634) | 0.004 |  |  |
| Alcoholic cirrhosis |  |  |  |  |
| No | 1 |  |  |  |
| Yes | 1.076 (0.586, 1.976) | 0.814 |  |  |
| **Smoking status** |  |  |  |  |
| Never smoker | 1 |  |  |  |
| Ever-smoker | 1.708 (1.058, 2.757) | 0.029 |  |  |
| **Alcohol use** |  |  |  |  |
| Never drink | 1 |  |  |  |
| Ever drink | 3.088 (1.836, 5.193) | < 0.001 |  |  |

**Supplementary Table 3** Factors associated with treatment assignment: Univariate and multivariate logistic regression (continue)

| **Covariables** | **Univariate analysis** | | **Multivariate analysis** | |
| --- | --- | --- | --- | --- |
|  | **OR (95% CI)** | **P-value** | **OR (95% CI)** | **P-value** |
| **BCLC stage at diagnosis** |  |  |  |  |
| A | 1 |  |  |  |
| B | 1.521 (0.603, 3.837) | 0.374 |  |  |
| C | 3.872 (1.598, 9.382) | 0.003 |  |  |
| **ECOG-PS** |  |  |  |  |
| 0-1 | 1 |  |  |  |
| 2-4 | 0.576 (0.165, 2.002) | 0.384 |  |  |
| **Child-Pugh classification** |  |  |  |  |
| A | 1 |  | 1 |  |
| Non-A | 5.912 (3.720, 9.393) | < 0.001 | 8.005 (3.649, 17.563) | < 0.001 |
| **MVI** |  |  |  |  |
| No | 1 |  |  |  |
| Yes | 1.653 (1.096, 2.492) | 0.016 |  |  |
| **EHS** |  |  |  |  |
| No | 1 |  |  |  |
| Yes | 0.942 (0.624, 1.422) | 0.775 |  |  |
| **Previous local treatment** |  |  |  |  |
| No | 1 |  |  |  |
| Yes | 0.249 (0.160, 0.386) | < 0.001 |  |  |
| **Laboratory** |  |  |  |  |
| **ALP** |  |  |  |  |
| <3x ULN | 1 |  |  |  |
| ≥3x ULN | 2.960 (1.566, 5.593) | 0.001 |  |  |
| **AST** |  |  |  |  |
| <3x ULN | 1 |  | 1 |  |
| ≥3x ULN | 2.486 (1.640, 3.770) | < 0.001 | 2.542 (1.215, 5.317) | 0.013 |
| **ALT** |  |  |  |  |
| <3x ULN | 1 |  |  |  |
| ≥3x ULN | 1.354 (0.626, 2.928) | 0.441 |  |  |

**Supplementary Table 3** Factors associated with treatment assignment: Univariate and multivariate logistic regression (continue)

| **Covariables** | **Univariate analysis** | | **Multivariate analysis** | |
| --- | --- | --- | --- | --- |
|  | **OR (95% CI)** | **P-value** | **OR (95% CI)** | **P-value** |
| **Creatinine, mg/dl** |  |  |  |  |
| <1.5 | 1 |  |  |  |
| ≥1.5 | 0.494 (0.145, 1.678) | 0.258 |  |  |
| **Hemoglobin, g/dl** |  |  |  |  |
| <8.5 | 1 |  |  |  |
| ≥8.5 | 2.700 (0.334, 21.823) | 0.351 |  |  |
| **WBC, /mm^3^** |  |  |  |  |
| <4000 | 1 |  |  |  |
| ≥4000 | 1.636 (0.706, 3.789) | 0.251 |  |  |
| **Platelet, /mm^3^** |  |  |  |  |
| <75,000 | 1 |  |  |  |
| ≥75,000 | 3.875 (0.900, 16.681) | 0.069 |  |  |
| **AFP, ng/ml** |  |  |  |  |
| <400 | 1 |  |  |  |
| ≥400 | 2.134 (1.353, 3.367) | 0.001 |  |  |

AFP, Alpha-fetoprotein; ALP, Alkaline Phosphatase; ALT, Alanine aminotransferase; AST, Aspartate aminotransferase; BCLC, Barcelona Clinic Liver Cancer; CI, confidence interval; CM, Maharaj Nakorn Chiang Mai hospital; CSMBS, Civil Servant Medical Benefit Scheme; ECOG-PS, Eastern Cooperative Oncology Group performance status; EHS, extrahepatic spreading; HBV, hepatitis B virus; HCV, hepatitis C virus; LPCH, Lampang Cancer hospital; MVI, major vascular involvement; OR, Odds ratio; RAMA, Ramathibodi hospital; SSS, Social Security Scheme; UC, universal health coverage scheme; ULN, upper limit of normal; VH, Vajira hospital; WBC, White Blood Cell

**Supplementary Table 4** Response to treatment

| **Response** | **Total**  (n=504) | **Sorafenib**  (n=382) | **FOLFOX**  (n=122) |
| --- | --- | --- | --- |
| CR | 2 (0.4) | 2 (0.5) | - |
| PR | 24 (4.8) | 18 (4.7) | 6 (4.9) |
| SD | 119 (23.6) | 99 (25.9) | 20 (16.4) |
| PD | 210 (41.7) | 166 (43.5) | 44 (36.1) |
| NE | 149 (29.5) | 97 (25.4) | 52 (42.6) |

Value are expressed as n (%)

CR, complete response; FOLFOX, Fluorouracil, Leucovorin, and Oxaliplatin; NE, not evaluate; PD, progression of disease; PR, partial response; SD, stable disease


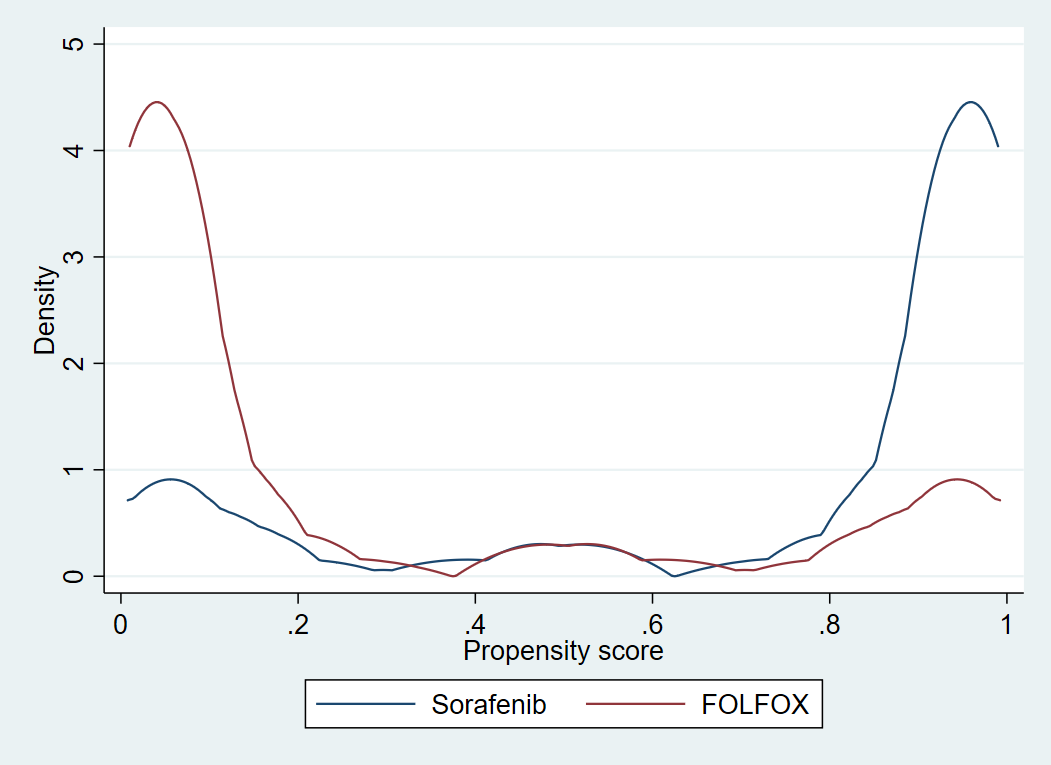


**Supplementary Figure 1** Overlapping plot


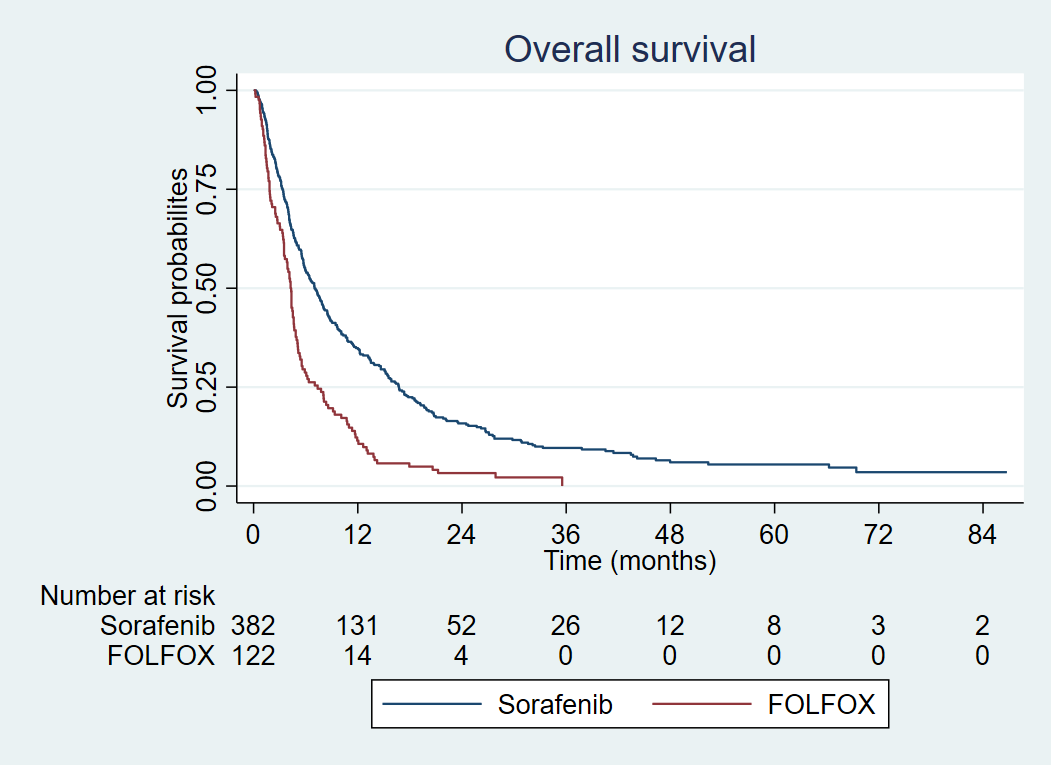


| **Treatment** | **Median OS (95% CI) (months)** |
| --- | --- |
| Sorafenib | 7.02 (5.93-8.07) |
| FOLFOX | 4.26 (3.51-4.62) |

**Supplementary Figure 2** Kaplan-Meier survival curve for overall survival by treatment regimens


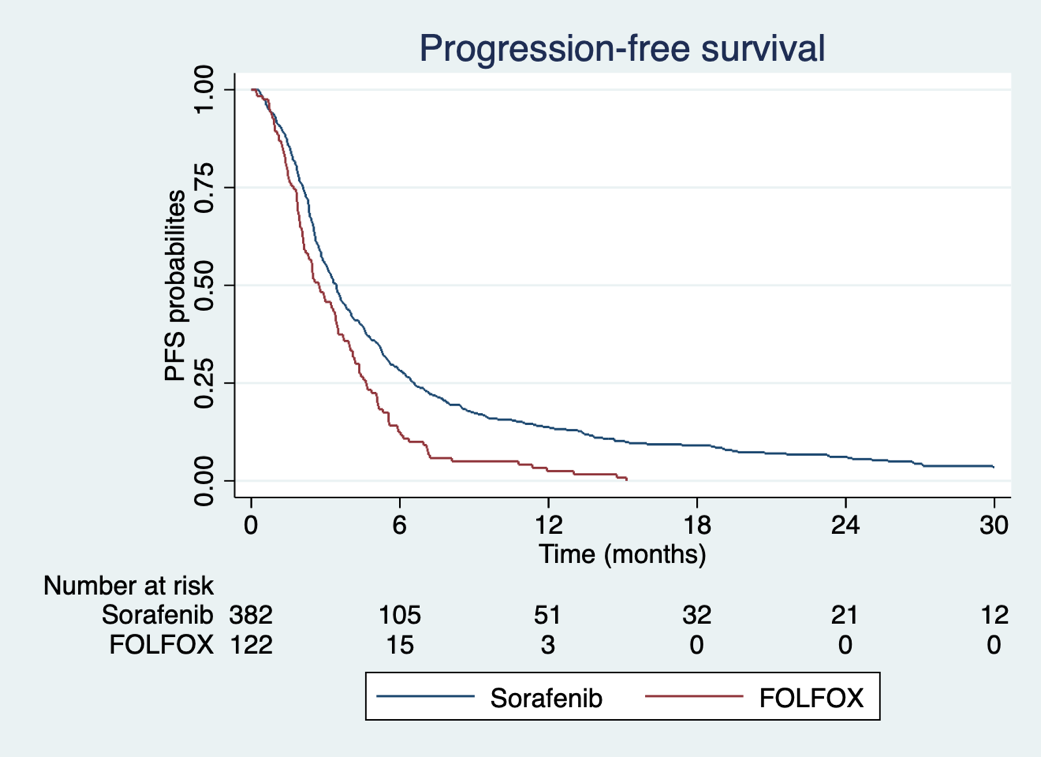


| **First line treatment** | **Median PFS (95% CI) (months)** |
| --- | --- |
| Sorafenib | 3.4 (3.0, 3.8) |
| FOLFOX | 2.7 (2.1, 3.4) |

**Supplementary Figure 3** Kaplan-Meier survival curve for progression-free survival

by treatment regimens
